# Niche derived oligodendrocyte progenitors: a source of rejuvenation or complementation for local oligodendrogenesis?

**DOI:** 10.3389/fncel.2013.00188

**Published:** 2013-10-22

**Authors:** Sylvia Agathou, Ragnhildur T. Káradóttir, Ilias Kazanis

**Affiliations:** Department of Veterinary Medicine, John van Geest Centre for Brain Repair, Wellcome Trust-MRC Stem Cell Institute, University of CambridgeCambridge, UK

**Keywords:** oligodendrocyte precursor cell (OPC), Myelination, subventricular zone (SVZ), subependymal zone, glia cell, stem cell, CNS

**Figure 1:**
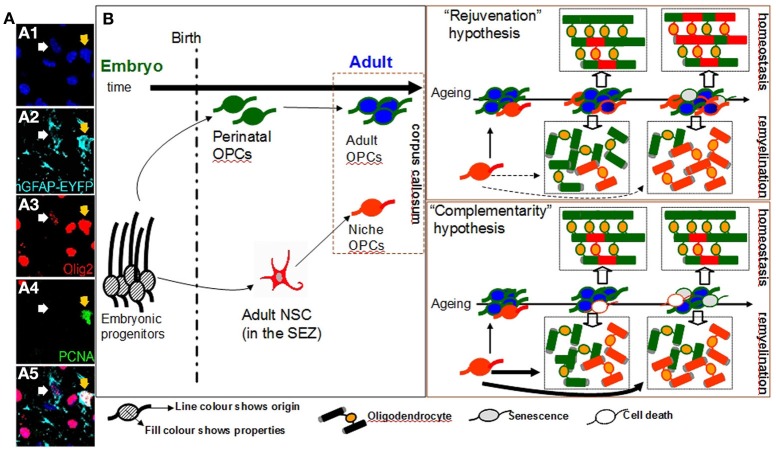
**Oligodendrogenesis from niche and parenchymal progenitors in the adult corpus callosum.** (Panel **A**) Numerous cells of the oligodendroglial lineage (both OPCs and oligodendrocytes, here all identified by expression of Olig2, in red) populate the adult corpus callosum (cc). A fraction of these is generated within the adjacent SEZ cytogenic niche (here identified by the expression of EYFP, in cyan, with two examples shown with arrows). OPCs are a mitotically active cell population as indicated by the expression of the mitotic marker PCNA (in green; note that the yellow arrow shows a proliferating OPC of SEZ origin). (Panel **B**: Diagram) A schematic illustration of the possible role of niche-derived OPCs in oligodendrogenesis in the adult cc. One fraction of OPCs in the cc is derived from perinatal OPCs (cells of green outline) that have switched to an adult, self-renewing mode of division (cells of blue fill); whilst, another is derived from adult neural stem cells located in the SEZ (cells of red outline). According to the “rejuvenation hypothesis”, niche OPCs migrate in the cc and gradually acquire properties similar to those of parenchymal adult OPCs (such as self-renewing potential). According to this scenario the main role of niche OPCs is to provide freshly-born self-renewing progenitors, thus partially compensating for the increase in the numbers of adult parenchymal OPCs that show signs of senescence over ageing (cells of gray fill). These niche OPCs contribute to the constant remodeling of myelin during homeostasis and to the generation of oligodendrocytes after demyelinating insults (such as during the course of diseases like multiple sclerosis). Within the ageing cc the contribution of the “younger” niche OPCs is increased (note the generation of higher numbers of red oligodendrocytes over time). According to the alternative “complementarity hypothesis”, the two sources of OPCs co-exist, albeit operating independently and with niche OPCs generating oligodendrocytes directly in response to local demand but without exhibiting long-term self-renewing capacity. In this scenario the contribution of niche-driven oligodendrogenesis is overall lower than that expected based on the “rejuvenation hypothesis”, especially regarding the homeostatic myelin remodeling. [Images in panel **A** show immunostained adult mouse brain cryosections. The double transgenic mice hGFAP-Cre^ERT2^ x Rosa26-EYFP were used. In these mice EYFP expression is induced in neural stem cells of the SEZ and is maintained in all of their progeny].

With the recent revelations that strikingly plastic cytogenic and migratory processes take place in the adult mammalian brain, it cannot anymore be considered as an organ static or utterly unequipped against injury. The Subependymal Zone (SEZ) or otherwise called with its embryonic equivalent as the subventricular zone, is one major cytogenic area located at the lateral wall of the lateral ventricles (Luskin, 1993) and is known to continuously contribute new cells to different brain areas both during homeostasis and following injury (Kazanis, 2009). A well-documented role of the SEZ in rodents is the constant supplementation of neural progenitors to the olfactory bulb (OB), through the rostral migratory stream (Lois and Alvarez-Buylla, 1994). Once in the OB, SEZ-derived neural progenitors differentiate into GABAergic (mainly) and glutamatergic neurons, depending on their exact anatomical origin in the niche (Brill et al., 2009). Cellular integration and survival depends on sensory olfactory inputs (i.e., exposure to new odors), as well as on the ability of these neural progenitors to form the appropriate synaptic connections with other cells (Mouret et al., 2008). This supply of new neurons to the OB is not only necessary for olfactory learning and memory but also for successful social and mating encounters in rodents (Oboti et al., 2011). Despite its initially identified neurogenic output, the SEZ is now known to also generate oligodendrocyte progenitor cells (OPCs) from Olig2-expressing transit-amplifying progenitors in the niche (TaPs/ also known as type C cells). During early post-natal stages stem and progenitor cells of the SEZ generate oligodendrocyte progenitors that migrate in a wide range of brain areas, including the corpus callosum, the cerebral cortex and the striatum, where they give rise to myelinating oligodendrocytes (Zerlin et al., 1995). After the SEZ assumes its mature structure (Alves et al., 2002) and switches to an adult phenotype (Jablonska et al., 2007), the adult niche-derived OPCs seem to migrate shorter distances, such as to the closely located corpus callosum (Cayre et al., 2006; Menn et al., 2006) (Figure 1), but in response to demyelination can migrate further afield such as to the striatum (Capilla-Gonzalez et al., 2013b). However, currently little is known about the characteristics of niche-derived adult OPCs and the functions they might serve.

In contrast to the less studied SEZ-derived adult OPCs, much more is known about adult parenchymal OPCs. During perinatal development OPCs are generated in different waves (Kessaris et al., 2006), firstly from sonic hedgehog (shh)-responsive ventral progenitors and at later stages from dorsal progenitors in a shh-independent manner (Fogarty et al., 2005). Subsequently, perinatal OPCs migrate away to populate the gray and white matter and to give rise to the myelinating cells of the central nervous system, the oligodendrocytes. Once early post-natal myelination program is concluded, some OPCs remain to give rise to the parenchymal adult OPCs which are equally distributed around the brain and account for a striking 5% of the total cell population with the majority of them (>80%) being mitotically active (Polito and Reynolds, 2005; Young et al., 2013). These parenchymal OPCs are capable of responding to demyelinating insults by quickly migrating to the injured site where they proliferate and give rise to new myelinating oligodendrocytes, a process called remyelination (Franklin and Ffrench-Constant, 2008). In light of the abundance of adult parenchymal OPCs and their capacity to successfully replace damaged oligodendrocytes following injury, the role of adult SEZ-derived OPCs in the brain remains a puzzle, especially because there is no evidence to support that adult niche and parenchymal OPCs behave in any significantly different way (Caillava et al., 2011).

One approach to address this puzzle is to look into the development of adult parenchymal OPCs. Experiments performed in the late 80's suggested that adult OPCs originate from a fraction of the late-embryonic and perinatal OPC population (Zerlin et al., 1995) that escapes terminal differentiation to oligodendrocytes (Wren et al., 1992). Over time, these perinatal OPCs switch to a significantly different adult-like behavior with an almost 4-fold slower cell cycle as compared to perinatal OPCs (Shi et al., 1998; Tang et al., 2000; Young et al., 2013). Moreover, adult and perinatal parenchymal OPCs respond differently to growth factors such as platelet derived growth factor (PDGF) and neuregulin *in vitro* (Shi et al., 1998); downregulate membrane ion channel expression (Clarke et al., 2012); and have different migratory and remyelinating properties (Windrem et al., 2004). With ageing, increasing numbers of adult parenchymal OPCs start expressing various markers of senescence (Kujuro et al., 2010) and their efficiency in regenerating damaged oligodendrocytes significantly deteriorates (Sim et al., 2002). This phenomenon has been linked both with the deterioration of health in patients suffering from chronic demyelinating diseases such as multiple sclerosis (Franklin and Ffrench-Constant, 2008), but also with cognitive decline in the elderly (Sullivan et al., 2010). Therefore, one possible scenario is that, the main function of SEZ-derived OPCs is to replenish the ageing pool of adult parenchymal OPCs, with freshly-born self-renewing progenitors from adult neural stem cells. This “rejuvenating hypothesis”, according to which a functional convergence between adult niche and parenchymal OPCs exists, leads to a clear prediction, that the adult OPC pool in the supra-ventricular corpus callosum should gradually become dominated by self-renewing OPCs of SEZ origin. Recently, it was suggested that low-level oligodendrocyte turnover occurs under physiological conditions in the corpus callosum (a process named myelin remodeling)(Rivers et al., 2008; Clarke et al., 2012; Young et al., 2013); therefore, the numbers of oligodendrocytes generated by SEZ-derived progenitors in the homeostatic corpus callosum should also significantly increase over time. Notably, the contribution of SEZ-driven oligodendrogenesis should become more apparent in cases of extensive generation of oligodendrocytes, such as during remyelination (Figure 1).

An alternative scenario regarding the fate of SEZ-derived OPCs could be drawn by looking at the neurogenic output of the SEZ. Progenitors of neuronal commitment (neuroblasts) are generated within the niche by stationary adult neural stem cells and TaPs. Subsequently, they migrate in chains to the OB where they disperse radially to the different neuronal layers (Lois and Alvarez-Buylla, 1994; Brill et al., 2009), where a small fraction survives long-term by fully differentiating and integrating into existing networks, while the rest die. There is no evidence for long-term self-renewal potential of neuroblasts and the levels of SEZ-driven neurogenesis at the OB are controlled by the proliferative activity of stem cells into the distant niche, as well as by the rate of migration and differentiation/integration of the neuroblasts. Notably, cellular integration and survival of SEZ-derived neuroblasts depends on sensory olfactory activity. Long-term exposure to odor enriched environments or to odor discrimination learning protocols, dramatically increases the survival of SEZ-derived neuroblasts in the murine OB (Mouret et al., 2008; Oboti et al., 2011). This activity-dependent neurogenesis is also prominent in the other major stem cell niche of the adult brain, the subgranular zone (SGZ) of the dentate gyrus (DG). New neurons (but no OPCs) are constantly born in the SGZ and subsequently migrate and populate the granule cell layer of the DG where they differentiate into glutamatergic granule cells. It has been documented that various environmental cues ranging from voluntary exercise to environmental enrichment and training on associative tasks, significantly increase adult DG neurogenesis (Ma et al., 2009). If this scenario also applies to SEZ-driven oligodendrogenesis, then niche-derived OPCs enter the corpus callosum and either differentiate to mature oligodendrocytes or die, depending on local demands. According to this “complementarity hypothesis”, in which niche and parenchymal oligodendrogeneses co-exist but operate in an independent and supplemental way, the predictions would be i) a lack of SEZ-derived OPC accumulation within the pool of OPCs in the homeostatic adult corpus callosum (in contrast to the “rejuvenating hypothesis”), ii) a limited involvement of SEZ-generated oligodendrocytes to the suggested low-level homeostatic myelin remodeling (Clarke et al., 2012; Young et al., 2013), and iii) in cases of remyelination, a dramatically accelerated contribution of adult niche OPCs directly migrating by the niche (Figure 1). If this hypothesis is correct, then the overall oligodendrogenic output of the SEZ over time will be lower than that of the “rejuvenating hypothesis” due to the lack of contribution from corpus callosum self-renewing OPCs of niche origin. In support of this scenario in experimentally-induced corpus callosum demyelination, oligodendrogenesis is also driven by neuron-committed neuroblasts that are capable of escaping their route to the OB and of migrating toward the lesion where they switch cell fate and establish synaptic contacts with axons (Etxeberria et al., 2010) similarly to parenchymal OPCs during development (Káradóttir et al., 2005; Kukley et al., 2007; Ziskin et al., 2007). Subsequently they differentiate into myelinating oligodendrocytes with chordin and netrin1 having been identified as regulatory signals in this process (Jablonska et al., 2010; Cayre et al., 2013). This response-mediated oligodendrogenic plasticity of the SEZ has also been documented in experimental autoimmune encephalitis (an animal model of multiple sclerosis) with increased oligodendrogenesis from the niche in the expense of neurogenic fates (Tepavcevic et al., 2011). But if this is how SEZ-derived oligodendrogenesis occurs, does it actually present any advantages over the parenchymal oligodendrogensis?

As old age seems to be the major factor underlying the decreased efficiency of remyelination in the CNS (Sim et al., 2002; Hampton et al., 2012; Ruckh et al., 2012), the existence of an active neural stem cell population that can either constantly supply new-born OPCs (rejuvenating hypothesis), or can directly generate high numbers of oligodendrocytes upon demand (complementary hypothesis), provides a valuable explanation for SEZ-driven oligodendrogenic potential (see the ageing scenarios in Figure 1). Notably, neural stem cells located in the SEZ retain their differentiation potential during ageing (Ahlenius et al., 2009; Capilla-Gonzalez et al., 2013a in this volume of FCN) and increase their homeostatic mitotic activity to compensate for the gradual depletion of their population (Shook et al., 2012), properties that confer to the niche a level of resistance to ageing. Significant questions remain to be addressed concerning the exact role of SEZ-driven oligodendrogenesis under physiological and pathological conditions. It is certainly possible that the role of the SEZ falls into both the above-stated hypotheses with not only a role in rejuvenating the adult parenchymal pool of OPCs, but also constantly responding to various environmental cues by providing a cellular boost to the areas in need. There is a definite necessity for a better understanding of adult human oligodendrogenesis and neurogenesis and whether age-related brain pathology is strongly linked to defects in the neurogenic niches.
